# Primary breast lymphoma: Patient profile, outcome and prognostic factors. A multicentre Rare Cancer Network study

**DOI:** 10.1186/1471-2407-8-86

**Published:** 2008-04-01

**Authors:** Wendy Jeanneret-Sozzi, Alphonse Taghian, Ron Epelbaum, Philip Poortmans, Daniel Zwahlen, Beat Amsler, Sylviane Villette, Yazid Belkacémi, Tan Nguyen, Pierre Scalliet, Philippe Maingon, Cristina Gutiérrez, Pauline Gastelblum, Marco Krengli, Rita Abi Raad, Mahmut Ozsahin, René-Olivier Mirimanoff

**Affiliations:** 1 Centre Hospitalier Universitaire Vaudois and University of Lausanne, Rue du Bugnon, CH-1011 Lausanne, Switzerland; 2 Massachusetts General Hospital, 32 Fruit Street, Boston, MA 02114, USA; 3 Rambam Medical Centre, PO Box 9602, Haifa 31096, Israel; 4 Dr. Bernard Verbeeten Instituut, Brugstraat, NL-5000 LA Tilburg, The Netherlands; 5 Universitätsspital, Rämistrasse 100, CH-8091 Zürich, Switzerland; 6 Universitätskliniken, Petersgraben 4, CH-4031 Basel, Switzerland; 7 Centre Oscar Lambret, 3 rue F. Combémale, F-59000 Lille, France; 8 Hôpital Tenon, 4 Rue Chine, F-75020 Paris, France; 9 Centre Jean Godinot, BP 171, F-51100 Reims, France; 10 Cliniques Universitaires St. Luc, Ave. Hippocrate Brussels, Belgium; 11 Centre Georges François Leclerc, Rue du Professeur Marion, F-21034 Dijon, France; 12 Institut Català d'Oncologia, Av. Gran Via s/n km.2.7, E-08907 L'Hospitalet, Barcelona, Spain; 13 Institut Jules Bordet, 1 rue Héger Bordet, B-1000 Brussels, Belgium; 14 Ospedale Maggiore della Carità, Università del Piemonte Orientale, I-28100 Novara, Italy

## Abstract

**Background:**

To asses the clinical profile, treatment outcome and prognostic factors in primary breast lymphoma (PBL).

**Methods:**

Between 1970 and 2000, 84 consecutive patients with PBL were treated in 20 institutions of the Rare Cancer Network. Forty-six patients had Ann Arbor stage IE, 33 stage IIE, 1 stage IIIE, 2 stage IVE and 2 an unknown stage. Twenty-one underwent a mastectomy, 39 conservative surgery and 23 biopsy; 51 received radiotherapy (RT) with (n = 37) or without (n = 14) chemotherapy. Median RT dose was 40 Gy (range 12–55 Gy).

**Results:**

Ten (12%) patients progressed locally and 43 (55%) had a systemic relapse. Central nervous system (CNS) was the site of relapse in 12 (14%) cases. The 5-yr overall survival, lymphoma-specific survival, disease-free survival and local control rates were 53%, 59%, 41% and 87% respectively. In the univariate analyses, favorable prognostic factors were early stage, conservative surgery, RT administration and combined modality treatment. Multivariate analysis showed that early stage and the use of RT were favorable prognostic factors.

**Conclusion:**

The outcome of PBL is fair. Local control is excellent with RT or combined modality treatment but systemic relapses, including that in the CNS, occurs frequently.

## Background

The term "primary breast lymphoma" (PBL) is used to define malignant lymphoma primarily occurring in the breast in the absence of previously detected lymphoma localizations.

The breast is a rare site for non-Hodgkin's lymphoma (NHL) and represents only between 0.38 and 0.7% of all NHL, and between 1.7 and 2.2 of all extranodal NHL [1-5], accounting for only 0.04 to 0.5% of all breast malignancies [2,3,5-10]. With the exception of the recently published prospective Mexican trial which included 96 patients with PBL [11] and the large study of the International Extranodal Lymphoma Study Group (IELSG) [12], we were able to find in the literature only retrospective studies with a relatively limited number of patients, often mixed with cases of secondary breast involvement, single case reports and clinicopathologic studies, the latter often lacking any follow-up information.

So far, the largest retrospective series with genuine PBL and sufficient follow-up identified 204 patients from the IELSG [12]. Other studies included between 20 and 53 cases and provide some interesting information [3,6,13-20]. However, because of the limited number of patients and the sometimes extended time span, the parameters of this disease, such as natural history, prognostic factors, impact of treatment, patterns of failure and survival have not been always well identified. For this reason and to collect a larger number of patients affected by this rare entity, we have performed a multicenter international retrospective analysis on PBL within the Rare Cancer Network [21]. The purpose of this study was to better assess the clinical profile, treatment parameters, patterns of failure, survival, and prognostic factors in patients presenting with PBL. This report includes 84 patients with PBL.

## Methods

### Patient characteristics

Eighty-four patients with primary breast lymphoma (PBL) were evaluated and treated in 20 member institutions of the Rare Cancer Network between 1970 and 2000. By definition, all cases presented with a lymphomatous involvement of the breast as the first manifestation of their disease with no previous diagnosis of NHL of any type or site.

The 84 patients included 83 women (99%) and 1 man. The median age was 64 years (range 28–90 years). The median follow-up was 56 months (range 9–188 months). Five patients presented with a previous cancer in the following sites: uterus (2), tonsil (1), kidney (1), bladder (1), and 2 had a concomitant cancer: of the breast (1) and soft tissue sarcoma of the leg (1). All were pathology-confirmed NHL, 46 with stage IE, 33 with stage IIE, 1 with stage IIIE and 2 with stage IVE, according to the Ann Arbor classification [22]. For 2 patients, the stage could not be retrieved. All patients received treatment with curative intent. The pathology reports were centrally reviewed. All NHL in this series were classified or reclassified according to the Working Formulation [23] as it was thought to be the most reproducible classification when reclassifying cases which were included in this 30-year period and coming from 20 different institutions. Thus, 51 patients (61%) had a high-grade, 6 (7%) an intermediate grade, and 27 (32%) a low-grade NHL.

For all patients, data on the medical history and physical examination were available. In fifty-one patients the initial sign was a palpable mass, in 9 there were local inflammatory signs, 10 had pain, 21 had palpable lymph nodes, and in 10 patients the PBL was discovered by a routine mammography. Staging work-up for all patients is shown in detail in Table 1, while patient characteristics are summarized in Table 2.

**Table 1:** Staging work-up in 84 patients with primary breast lymphoma

Staging work up	n	%
Mammography	65	77
Mammary ultra sound	25	30
Mammary MRI	7	8
Chest x-ray	72	86
Chest CT-scan	66	79
Abdominal CT-scan	70	83
Abdominal ultra sound	28	33
Bone marrow biopsy or aspiration	77	92
Lymphography	11	13
Bone scintigraphy	17	20
CSF examination	14	17
CBC	82	98
ESR	67	80
Serum protein electrophoresis	52	62
B_2 _microglobulin	39	46

MRI: magnetic resonance imaging

CSF: cerebrospinal fluid

CBC: complete blood count

ESR: erythrocyte sedimentation rate.

**Table 2:** Patient characteristics

**Characteristic**	**N**	**%**
Gender		
- male	1	1
- female	83	99
Symptoms and signs		
- local pain	10	12
- local inflammation	9	11
- palpable mass	51	61
- palpable lymph nodes	21	25
- incidental mammography finding	10	12
Localization (71 patients)		
- external superior	34	48
- internal superior	10	14
- external inferior	9	13
- internal inferior	5	7
- central	12	17
- entire breast	1	1
Tumor size (71 patients)		
- 0 – 1.9 cm	4	6
- 2 – 4.9 cm	35	49
- 5 – 9.9 cm	23	32
- 10 – 15 cm	9	13
Stage (82 patients)		
- I E	46	55
- II E	33	39
- III E	1	1
- IV E	2	2
- unknown	2	2
Grade (according to Working Formulation)		
- high	51	61
- intermediate	6	7
- low	27	32

### Surgery

Surgery consisted of mastectomy with (16) or without (5) axillary dissection, quadrantectomy alone (3), tumorectomy with (1) or without (35) axillary dissection, and biopsy alone (23). In one case, the type of surgery could not be identified. The majority of patients received post-operative treatment: 37 radiotherapy combined with chemotherapy, 22 chemotherapy only, 14 radiotherapy only, whereas 11 patients had no treatment after surgery. The reasons for not giving any adjuvant treatment were patients' refusal and age in 2. For the remainder, the reasons could not be retrospectively identified.

### Radiotherapy (RT)

Amongst the 51 patients (61%) receiving radiotherapy, 41 were treated on the whole breast, 7 on the thoracic wall, and 27 on the regional lymph nodes with or without breast/chest wall irradiation. The median RT dose was 40 Gy (range 12–55 Gy) at a median daily dose of 2 Gy (range 1.8–3 Gy). Except for 1 patient treated with electrons on the thoracic wall, all breasts or thoracic walls were treated with tangential photon fields.

### Chemotherapy

Fifty-nine of the 84 patients (70%) were treated with chemotherapy. For 25 of them, chemotherapy consisted of CHOP (i.e. cyclophosphamide, doxorubicin, vincrinstine and prednisone). Ten patients received a CHOP-like regimen (i.e. cyclophosphamide, epirubicin, vincristine and prednisone), 11 CHOP plus bleomycine and 2 CHOP plus methotrexate. Three patients received a CVP regimen (cyclophosphamide, vincristine and prednisone) and 3 chlorambucil alone. Five patients received other chemotherapy drugs, including various combinations of cyclophosphamide, mitoxantrone, VM-26, doxorubicin, and prednisone. The median number of chemotherapy cycles was 4 (range 1–12). Only one patient received one cycle and 2 patients 2 cycles. This was due to haematological toxicity 2 or non-haematological toxicity 1.

### Statistical Analysis

Overall survival (OS), lymphoma-specific survival (LSS), disease-free survival (DFS), and local control (LC) were calculated from the day of histology examination using the Kaplan-Meier method (243). The events were death (including all causes of death) for OS, death or relapse for DFS, death from lymphoma for LSS and local relapse for local control. Differences between groups were assessed using the log-rank test [25]. We screened for independent prognostic factors with a Cox regression analysis [26]. A p-value of < 0.05 was considered statistically significant.

## Results

### Local and systemic relapse

Following treatment, 10 of the 84 patients (12%) presented with a local relapse. The median time to local relapse was 17 months (range 2–61 months).

Systemic lymphoma relapse, with or without local relapse, was observed in 43 of 84 patients (55%). The sites of the first systemic relapses were deep-seated (thoracic and abdominal) lymph nodes (n = 13), peripheral lymph nodes (n = 8), central nervous system (CNS) (n = 12), bone marrow (n = 7), pleura (n = 5), skin (n = 4), lungs (n = 3), spleen, muscles, orbit, stomach, Waldeyer's ring and kidney (each n = 2) and liver (n = 1). Of the 12 patients with CNS relapse, 10 or 85% had a high grade lymphoma, versus 61% for the entire group of patients. The median time to systemic relapse was 22 months (range 4–140).

### Survival

**Figure 1:**
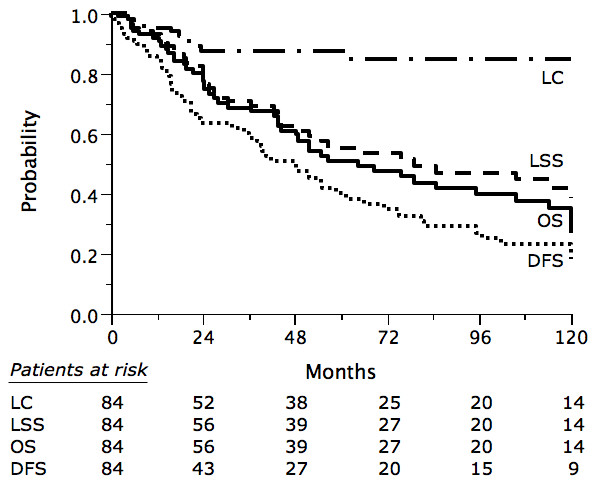
Overall survival (OS), lymphoma-specific survival (LSS), disease-free survival (DFS) and local control (LC) in 84 patients with primary breast lymphoma.

The 5-yr OS, LSS, DFS and LC were 53%, 59%, 49%, and 87% respectively (Fig. 1). Thirty-four patients died due to PBL and 8 due to other causes (1 from metastatic lung cancer, 1 from septicemia, 1 from small bowel obstruction, 1 from liver insufficiency, and for 4 patients the cause of death could not be retrieved).

### Prognostic factors

Univariate analyses revealed that early (IE) stage was a statistically significant favorable prognostic factor for OS, LSS, DFS and LC. Mastectomy was an adverse factor for OS and LSS, whereas RT was a statistically significant favorable prognostic factor for LSS and LC. Chemotherapy only marginally improved the LC but without reaching a significant level. Combined modality had only a positive and significant effect on local control (Table 3).

**Table 3:** Univariate analysis (logrank test)

	n	5 y OS (%)	P value	5 yr LSS (%)	P value	5 y DFS (%)	P value	5 yr LC (%)	P value
All patients	84	53		59		41		87	
Age (yrs)									
• <64	42	60	0.07	67	NS	45	NS	92	NS
• >64	42	44		49		38		82	
Stage									
• I E	46	62	0.007	70	0.006	52	0.05	92	0.05
• II E	33	40		44		28		81	
Grade									
• High	51	42	NS	50	NS	36	NS	85	NS
• Low/intermediate	33	66		70		48		90	
Tumor size									
• < 4 cm	38	58	NS	68	NS	40	NS	91	NS
• > 4 cm	33	43		44		43		83	
Surgery									
• C.S.	63	62	0.03	69	0.01	48	NS	91	NS
• Mastectomy	21	22		26		18		78	
RT									
• Yes	51	58	NS	70	0.02	41	NS	95	0.005
• No	33	46		46		40		76	
Chemotherapy									
• Yes	59	47	NS	51	NS	39	NS	92	NS
• No	25	61		72		44		77	
Combined modality									
• Yes	37	54	NS	64	NS	37	NS	96	0.03
• no	47	51		56		43		81	

In multivariate analysis, early stage was a significant favorable prognostic factor for OS, LSS, DFS, and LC (p values: 0.002, 0.001, 0.04, 0.03, respectively). Tumor size of more than 4 cm was an adverse prognostic factor for OS and LSS (p values: 0.01 and 0.007 respectively). RT was a favorable factor for local control (p = 0.02).

### Side effects of RT

Sixteen patients were reported to have grade 1–2 skin reactions, 2 presented with grade 2 esophagitis, and 2 with local edema. Only 1 patient developed grade III toxicity, consisting of radiation-induced pulmonary toxicity.

## Discussion

To our knowledge, our series of patients collected in 20 institutions of the Rare Cancer Network is the second largest retrospective analysis of PBL, with 84 patients treated over a 30 year period. We have identified one recent prospective trial from Mexico with a larger number of cases, including 96 patients with PBL [11] and the large series of the IELSG with 204 patients [12]. Other studies with more than 20 patients with PBL were reported by investigators from the MSKCC (53 cases), Japan (48 cases), City of Hope (41 cases), Stanford (37 cases), Harvard (32 cases), The Mayo Clinics (26 cases), The Netherlands (25 cases), MDACC (23 cases), France (22 cases), and Institut Gustave Roussy (20 cases) [3,6,13-20]. Some of these reports have also included secondary breast lymphoma [3,6,14,15] and in one of them no information on follow-up was provided [3].

### Patient characteristics and pathology

In our study, the median age was 64 years, which is comparable to the 60–65 year range published by other authors [11-16]. Aggressive PBL has sometimes been associated with younger age [5,14]. PBL is extremely rare in males, with only one of our 84 patients, 1 of 23 patients from MDACC, 1 of 25 from the Mayo Clinic, 1 of 18 from St. Cloud [16,17,27], and none in the other series [11,13-15].

In the present experience, the most prominent initial signs were a tumor mass, a mass with local inflammation, and palpable lymph nodes. In the other series, a painless mass was the most commonly presenting sign in 80–100% of cases [13,16,28]. Ten of our patients (12%) were diagnosed by a routine mammography, which is higher than in other series [14]. A second preceding (n = 5) or synchronous (n = 2) cancer was present in 7 (8%) of our patients whereas in the MSKCC series, 17% of patients presented with a second cancer [13].

According to the Working Formulation, 61% of our patients had a high-grade, 7% an intermediate grade, and 32% a low-grade PBL. A predominance of diffuse large cell PBL was a constant feature in the IELSG, the Dutch, the MSKCC, the MDACC and the City of Hope pathology reviews, whereas the proportion of low or intermediate grade PBL was variable [3,12,13,15,16]. According to the comprehensive literature review by Brogi and Harris, the majority of PBL are diffuse large B-cell lymphoma (40 – 70%), MALT-type has a reported incidence of 0 – 44%, whereas the rare Burkitt's NHL can bilaterally involve the breasts of young, pregnant or lactating women with very aggressive clinical behavior [5].

### Diagnostic workup

For all patients, a medical history and physical examination were carried out. Seventy-seven percent of our patients underwent a mammography, 30% breast US and 8% breast MRI. Of interest, only 85% of the mammograms and 72% of the ultrasound examinations were interpreted as abnormal. In contrast to this, in the Harvard series, mammograms done after physical detection of a mass identified a parenchymal abnormality in all but one patient [14].

### Treatment

#### Surgery

Strikingly, mastectomy was associated with a poorer survival in the univariate analysis, compared with conservative procedures or biopsy. A higher risk of failures with radical surgery was also noted in Fruchart et al's report [18] and an adverse effect on cancer-specific survival was seen in the IELSG series [12]. Although the adverse effect of mastectomy can be influenced by other confounding factors, radical surgery is at best unnecessary and should be avoided in PBL. Ideally surgery should be limited to a biopsy to establish the correct histological diagnosis, leaving the treatment with curative intent to radiotherapy and chemotherapy [11-13,18,19,29].

#### Radiation Therapy

Fifty-one (61%) of our patients received radiotherapy, with or without chemotherapy. The median radiotherapy dose was 40 Gy (range 12–55 Gy) at a median daily dose of 2 Gy. No dose effect relationship could be found. In addition, we were not able to demonstrate an impact of elective nodal irradiation. The majority (8 of 10) of the patients with a local relapse, did not receive post-operative radiotherapy. Thus, radiotherapy to the breast or to the thoracic wall had a statistically significant positive impact on local control, with 95% vs. 76% 5-yr local control rate (p = 0.02). In their series of 19 patients treated with definitive RT at MSKCC, De Blasio et al. found a local control rate of 78% [26]. The positive role of radiotherapy was also suggested in other series [12,20,28,30]. These and our results confirm the central role of radiotherapy in PBL.

#### Chemotherapy

Chemotherapy was associated with a non-significant trend towards a better local control rate, (92% vs. 77% at 5 yrs). No effect could be demonstrated on overall or lymphoma-specific survival. In most reports, patients with intermediate or high grade PBL have received adjuvant chemotherapy [12,14,16,19,27,28,30-34]. Because of the high proportion of patients receiving chemotherapy in these series, it is difficult to define its precise role, although a positive impact on local control [30] and relapse [15] is suggested. In the IELSG series, anthracyclin-based chemotherapy was associated with higher overall survival [12]. In low-grade lymphoma, the role of adjuvant chemotherapy is very doubtful.

In addition to these various systemic treatments, newer therapies, such as rituximab [35,36] deserve further investigation, but experience in PBL is still limited [37].

#### Combined modality treatment

Thirty-seven (44%) of our patients were treated with combined modality treatment. A favorable impact on local control was observed (p = 0.03) in the univariate analysis which was not confirmed in the multivariate analysis. Aviles et al have recently published their randomized trial of PBL in which 96 patients were allocated to radiotherapy (n = 30), chemotherapy (n = 32), and combined modality treatment (n = 34) [11]. All were staged IE or IIE PBL, with a good balance of prognostic factors between the 3 treatment groups. At 10 years, actuarial overall survival was 50%, 50% and 76% (p < 0.01) respectively [11]. A positive impact of combined modality in PBL is also suggested is several retrospective series [12,15-17,19,33].

### Overall prognosis

With regard to the outcome of patients with PBL, overall prognosis was only fair, with an overall 5 yr survival rate of 53%. Even for stage IE, 5-yr overall survival was only 62%. The 5-year survival reported in other series varied from 50 – 82% [11,12,16,17,20,30,33] and was likely to be related to the distribution of prognostic factors in the different series.

### Local control

The local control rate in our series was 87% at 5 years. Unfortunately, information on local control in most other series is vary scarce. DeBlasio et al report a 78% local control rate [29] and in Dao et al's series only 1 of 13 patients presented with a local relapse [30] whereas in Ganjoo's series no recurrence occurred in the involved breast [20]. In spite of these limited data, overall local control for patients receiving RT alone or combined with chemotherapy appears to be generally excellent.

### Systemic relapse

Forty-three patients (55%) suffered from a systemic relapse predominantly in deep-seated (n = 13), and peripheral (n = 8) lymph nodes, the central nervous system (n = 12), and bone marrow (n = 7), after a median follow-up of 22 months (range 4–140 months). The high rate of central nervous system relapses in PBL was also found in other studies [11,17,19,34], and some authors have raised the question of prophylactic central nervous system therapy [11,12,19,34].

### Prognostic factors

As shown in other studies of the Rare Cancer Network, the successful collection of data for rare cancers enabled us to define various prognostic factors. In univariate analyses, there was a borderline non-significant trend for a better 5-year survival in patients younger than 64 years. Stage IE was highly significantly better than stage IIE concerning overall, lymphoma-specific, disease-free survival, and local control. Concerning treatment, surgery had a statistically significant negative impact on overall and lymphoma-specific survival, and radiotherapy a statistically significant positive effect on lymphoma-specific survival and local control. Neither chemotherapy nor combined modality treatment significantly influenced OS, LSS, and DFS, whereas combined modality had a significant impact on local control.

In the multivariate analyses, early stage remained statistically significant for OS, LSS, DFS and LC, tumour size for OS and LSS, whereas RT was significant only for LC. Prognostic factors were also found in some of the other studies. In particular, Ann Arbor Stage [13,16,17], International Prognostic Index [12,16], and grade [13] had a positive impact on overall survival and disease-free survival.

## Conclusion

Primary breast lymphoma is a rare presentation of NHL. Therefore one should remain cautious in drawing strong conclusions. However, as we have analyzed one of the largest groups of patients with PBL, we can conclude that overall prognosis is only fair, that local control is excellent with RT or combined modality treatment but systemic relapses, including in the CNS, still occur frequently. The identification of several prognostic factors may be useful indicators regarding the overall management of PBL.

## Competing interests

The author(s) declare that they have no competing interests.

## Authors' contributions

WJS coordinated the study together with ROM. All authors provided data collected from patients treated in their centres. MO carried out the statistical analysis and WJS and ROM collaborated on the writing of the final paper which was seen by all authors and in particular reviewed by PP, AT, MK and DZ.

## Pre-publication history

The pre-publication history for this paper can be accessed here:



## References

[B1] Freeman C, Berg JW, Cutler SJ (1972). Occurrence and prognosis of extranodal lymphomas. Cancer.

[B2] Mattia AR, Ferry JA, Harris NL (1993). Breast lymphoma: a B-cell spectrum including low-grade B-cell lymphoma of mucosa-associated lymphoid tissue. Am J Surg Pathol.

[B3] Arber DA, Simpson JF, Weiss LM, Rappaport H (1994). Non-Hodgkin's lymphoma involving the breast. Am J Surg Pathol.

[B4] Topalovski M, Cristan D, Mattson JC (1999). Lymphoma of the breast: a clinicopathologic study of primary and secondary cases. Arch Pathol Lab Med.

[B5] Brogi E, Harris NL (1999). Lymphomas of the breast: pathology and clinical behavior. Semin Oncol.

[B6] Tanaka T, Hsueh CL, Hayashi K, Awai M, Nishihara K, Konaga E, Ishikawa J, Orita K (1984). Primary malignant lymphoma of the breast: with a review of 3 cases among Japanese subjects. Acta Pathol Jpn.

[B7] Dixon JM, Lumsden AB, Krajewski A, Elton RA, Anderson TJ (1987). Primary lymphoma of the breast. Br J Surg.

[B8] Cohen PL, Brooks JJ (1991). Lymphoma of the breast. Cancer.

[B9] Giandini R, Piccolo C, Rilke F (1992). Primary non-Hodgkin's lymphoma of the female breast. Cancer.

[B10] Bobrow LG, Richards MA, Happerfield LC, Diss TC, Isaacson PG, Lammie GA, Millis RR (1993). Breast lymphomas: a clinicopathologic review. Hum Pathol.

[B11] Aviles A, Delgade S, Nambo J, Neri N, Murillo E, Cleto S (2005). Primary breast lymphoma: results of a controlled clinical trial. Oncology.

[B12] Ryan G, Martinelli G, Kuper-Hommel M, Tsang R, Prumeri G, Yuen K, Roos D, Lennard A, Devizzi L, Cragg S, Hossfeld D, Pratt G, Dell'Olio M, Choo SP, Bociek RG, Radford J, Lade S, Gianni AM, Zucca E, Cavalli F, Seymour JF, International Extranodal Lymphoma Study Group (2008). Primary diffuse large B-cell lymphoma of the breast: prognostic factors and outcomes of a study by the International Extranodal Lyphoma Study Group. Ann Oncol.

[B13] Brustein S, Filippa DA, Kimmel M, Lieberman PH, Rosen PP (1987). Malignant lymphoma of the breast. A study of 53 patients. Ann Surg.

[B14] Domchek SM, Hecht JL, Fleming MD, Pinkus GS, Canellos GP (2002). Lymphomas of the breast: primary and secondary involvement. Cancer.

[B15] Kuper-Hommel MJJ, Snijder S, Jansen-Heijnen ML, Vrints LW, Kluin-Nelemans JC, Coebergh JW, Noordijk EM, Vreugdenhil G (2003). Treatment and survival of 38 female breast lymphomas: a population-based study with clinical and pathological reviews. Ann Hematol.

[B16] Ha CS, Dubey P, Goyal LK, Hess M, Cabanillas F, Cox JD (1998). Localized primary non-Hodgkin's lymphoma of the breast. Am J Clin Oncol.

[B17] Wong WW, Schild SE, Halyard MY, Schomberg PJ (2002). Primary non-Hodgkin's lymphoma of the breast: the Mayo Clinic experience. J Surg Oncol.

[B18] Fruchart C, Denoux Y, Chasle J, Peny AM, Boute V, Ollivier JM, Genot JY, Michels JJ (2005). High-grade primary breast lymphoma: is it a different clinical entity?. Breast Cancer Res Treat.

[B19] Ribrag V, Bibeau F, El Weshi A, Freyfer J, Fadd C, Cebotaru C, Laribi K, Fenaux P (2001). Primary breast lymphoma: a report of 20 cases. Br J Haematol.

[B20] Ganjoo K, Advani R, Mariappan MR, McMillan A, Horning S (2007). Non-Hodgkin's lymphoma of the breast. Cancer.

[B21] Rare Cancer Network. http://www.rarecancer.net.

[B22] Carbone PP, Kaplan HS, Mushoff K, Smithers DW, Tubiana M (1971). Report of the committee on Hodgkin's disease classification. Cancer Res.

[B23] The Non-Hodgkin's Lymphoma Pathologic Classification Project. National Cancer Institute sponsored study of classifications of non-Hodgkin's lymphomas (1982). Summary and description of Working Formulation for clinical usage. Cancer.

[B24] Kaplan ES, Meier P (1958). Non-parametric estimation from incomplete observations. J Am Stat Assoc.

[B25] Peto R, Pike MC, Armitage P, Breslow NE, Cox DR, Howard SV, Mantel N, McPherson K, Peto J, Smith PG (1976). Design and analysis of randomized clinical trials requiring prolonged observations of each patient. I. Introduction and design. Br J Cancer.

[B26] Cox DR (1972). Regression models and life tables. J Roy Stat Soc.

[B27] Vignot S, Ledoussal V, Nodiot P, Bourguingnat A, Janvier M, Mounier M, Chérel P, Floiras JL, Turpin F (2005). Non-Hodgkin's lymphoma of the breast: a report of 19 cases and a review of the literature. Clin Lymphoma.

[B28] Lyons J, Myles J, Pohlman B, Macklis RM, Crowe J, Crownover RL (2000). Treatment and prognosis of primary breast lymphoma: a review of 13 cases. Am J Clin Oncol.

[B29] DeBlasio D, McCormick B, Straus D, Smith M, Brustein S, Nisce L, Fuks Z (1989). Definitive irradiation for localized non-Hodgkin's lymphoma of the breast. Int J Radiat Oncol Biol Phys.

[B30] Dao AH, Adkins RB, Glick AD (1992). Malignant lymphoma of the breast: a review of 13 cases. Am Surg.

[B31] Ariad S, Lewis D, Cohen R, Bezwoda WR (1995). Breast lymphoma: a clinical and pathological review and 10-year treatment results. S Afr Med J.

[B32] Gholam D, Bibeau F, El Weshi, Bosq J, Ribrag V (2003). Primary breast lymphoma. Leuk Lymphoma.

[B33] Huang DZ, He XH, Yang S, Shi YK (2004). Clinical and pathological analysis of 15 cases of primary breast lymphoma. Ai Zheng.

[B34] Yamazaki H, Hanada M, Kitada M, Kuyama J, Sato T, Nishikubo M, Ishida T, Inoue T, Inoue T (2003). Four cases of central nervous system involvement of breast malignant lymphoma. Jpn J Clin Oncol.

[B35] Habermann TM, Weller EA, Morrison VA, Gascoyne RD, Cassileth PA, Cohn JB, Dakhil SR, Woda B, Fisher RI, Peterson BA, Horning ST (2006). Rituximab-CHOP versus CHOP alone or with maintenance rituximab in older patients with diffuse large B-cell lymphoma. J Clin Oncol.

[B36] Hiddemann W, Kneba M, Dreyling M, Schmitz N, Lengfelder E, Schmits R, Reiser M, Metzner B, Harder H, Hegewisch-Becker S, Fischer T, Kropff M, Reis HE, Freund M, Wörmann B, Fuchs R, Planker M, Schimke J, Eimermacher H, Trümper L, Aldaoud A, Parwaresch R, Unterhalt M (2005). Frontline therapy with rituximab added to the combination of cyclophosphamide, doxorubicin, vincristine and prednisone (CHOP) significantly improves the outcome for patients with advanced-stage follicular lymphoma compared with therapy with CHOP alone: results of a prospective randomized study of the German Low Grade Lymphoma Study Group. Blood.

[B37] Avilés A, Castañeda C, Neri N, Cleto S, Nambo MJ (2007). Rituximab and dose-dense chemotherapy in primary breast lymphoma. Haematologica.

